# A randomized controlled trial of long-acting muscarinic antagonist and long-acting β2 agonist fixed-dose combinations in patients with chronic obstructive pulmonary disease

**DOI:** 10.1186/s12890-021-01403-y

**Published:** 2021-01-13

**Authors:** Masato Muraki, Yuki Kunita, Ken Shirahase, Ryo Yamazaki, Soichiro Hanada, Hirochiyo Sawaguchi, Yuji Tohda

**Affiliations:** 1 Department of Respiratory Medicine and Allergology, Kindai University Nara Hospital, 1248-1 Otoda-cho, Ikoma, Nara 630-0293 Japan; 2 Department of Respiratory Medicine and Allergology, Kindai University Hospital, 377-2 Ohnohigashi, Osakasayama, Osaka 589-8511 Japan

**Keywords:** Glycopyrronium/indacaterol, Umeclidinium/vilanterol, Tiotropium/olodaterol, Quality of life, Inhaler device, COPD

## Abstract

**Background:**

In chronic obstructive pulmonary disease (COPD) patients, combination treatment with long-acting muscarinic antagonist (LAMA) and long-acting β2 agonist (LABA) increases forced expiratory volume in one second and reduces symptoms compared to monotherapy. In Japan, three different once-daily fixed-dose combinations (FDCs) have been prescribed since 2015, although a direct comparison of these FDCs has never been performed. The objective of the present study was to compare the effectiveness, preference, and safety of three LAMA/LABA FDCs—glycopyrronium/indacaterol (Gly/Ind), umeclidinium/vilanterol (Ume/Vil), and tiotropium/olodaterol (Tio/Olo)—in patients with COPD.

**Methods:**

We enrolled 75 COPD outpatients (male:female ratio, 69:6; 77.4 ± 6.9 years). A prospective, randomized, crossover study was conducted on three groups using three FDCs: Gly/Ind; Ume/Vil; and Tio/Olo. Each medication was administered for 4 weeks before crossover (total 12 weeks). After each FDC administration, a respiratory function test and questionnaire survey were conducted. A comparative questionnaire survey of all three LAMA/LABA FDCs was conducted after 12 weeks (following administration of final FDC).

**Results:**

No significant differences in COPD Assessment Test or modified Medical Research Council dyspnea questionnaire were reported in the surveys completed after each FDC administration; no significant differences in spirometric items were observed. In the final comparative questionnaire survey, patients reported better actual feeling of being able to inhale following Gly/Ind administration compared with Tio/Olo, although no significant differences in adverse events or other evaluations were reported.

**Conclusions:**

The three LAMA/LABA FDCs administered to COPD patients show similar effects and safety, although some minor individual preference was reported.

*Trial registration* This study retrospectively registered with the University Hospital Medical Information Network Clinical Trials Registry (number UMIN000041342, registered on August 6, 2020).

## Background

An inhaled bronchodilator is a drug that increases forced expiratory volume in one second (FEV1), and/or improves other spirometric variables. Inhaled bronchodilators are central pharmacological treatments for chronic obstructive pulmonary disease (COPD). In particular, long-acting muscarinic antagonists (LAMAs) and β2 agonists (LABAs) significantly improve respiratory function, dyspnea, and health status, and significantly reduce exacerbation rates [1]. Compared to LAMA or LABA monotherapy, or an Inhaled corticosteroid (ICS)/LABA combination, LAMA/LABA co-treatment is superior in several aspects, including in the improvement of symptoms [2–4] and respiratory functions [3–5], and in the prevention of exacerbations [6–8]. Therefore, LAMA and LABA co-treatment is recommended when the effect of a single bronchodilator is inadequate [1]. LAMA/LABA combination therapy using a single inhaler is recommended to enhance adherence, to reduce medical costs [5, 9, 10], and to optimize the synergistic effect of LAMA and LABA [11, 12].

As of September 2019, four LAMA/LABA fixed-dose combination (FDC) medicines—glycopyrronium/indacaterol (Bevespi®), glycopyrronium/indacaterol (Gly/Ind; Ultibro®), umeclidinium/vilanterol (Ume/Vil; Anoro®), and tiotropium/olodaterol (Tio/Olo; Spiorto®)—are available for patients with COPD in Japan. Only Gly/Ind, Ume/Vil, and Tio/Olo are administered once daily. Although an indirect comparison of these three medications has previously been reported [13], direct comparisons between any two of these medications are limited [14, 15], and no direct controlled study of three once-daily FDC medications has been reported. Therefore, a direct controlled comparison study with Gly/Ind, Ume/Vil, and Tio/Olo was conducted, and the effectiveness, preference, and safety of these three medications were compared and investigated.

## Methods

### Patients

COPD outpatients aged 40 years old or over who attended the Department of Respiratory Medicine and Allergology at Kindai University Nara Hospital (Ikoma, Japan) between April 2017 and October 2019, and who were diagnosed as requiring LAMA and LABA, were included in this study. Prior informed consent was obtained from all patients. The exclusion criteria were as follows: inability to inhale unassisted; inability to perform spirometry tests; pregnancy; severe comorbidities affecting quality of life, including malignancy, cardiac failure, renal failure, or severe liver dysfunction; and comorbidity of severe prostatic hypertrophy and closed-angle glaucoma. Patients with overlapping asthma could be enrolled, if the asthma presented as a stable disease without any exacerbations during the six months prior to the study.

### Study design

**Fig. 1:**
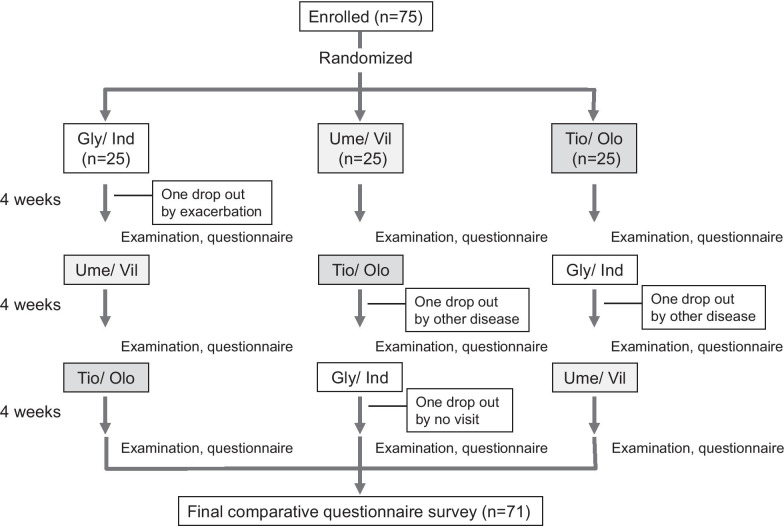
Trial profile

The open-labeled, prospective, randomized, crossover protocol used in this study is shown in Fig. 1. Subjects were randomly assigned to a Gly/Ind first group (Gly/Ind to Ume/Vil to Tio/Olo), an Ume/Vil first group (Ume/Vil to Tio/Olo to Gly/Ind), or a Tio/Olo first group (Tio/Olo to Gly/Ind to Ume/Vil) by the envelope method. Additional concomitant medications remained unchanged during the study. If ICS was used in an ICS/LABA combination, the dose of ICS monotherapy was adjusted to match the dose used in prior combination therapy. This study adheres to CONSORT guidelines.

### Interventions

The background details of included patients were examined and recorded. A pharmacist instructed patients within each group to inhale the prescribed medication once daily for 4 weeks. After 4 weeks (for each treatment regimen), the following items were evaluated: a COPD Assessment Test (CAT); a modified Medical Research Council dyspnea questionnaire (mMRC); an original questionnaire concerning side effects, effects, and the device (Additional file 1: Table S1); spirometry, using a Chestac-33 (Chest M.I., Tokyo, Japan); and, respiratory system resistance and reactance, determined via the forced oscillation technique (FOT) using a MostGraph-01VR (Chest M.I., Tokyo, Japan). If exacerbation due to common cold or other causes overlapped with the visit date, the evaluation appointment could be postponed for up to 4 weeks. The examinations were performed from 9:30 am to 11 am, 2.5–3.5 h after the patients took their normal morning medications (the LAMA/LABA combination and all other morning medications). Upon completing the final course (after 12 weeks), an original questionnaire survey with a ranking policy (Additional file 1: Figure S1) was conducted to compare the three medications. The relationship between first-rank medication selected and patient background factors was subsequently investigated.

### Statistical analyses

Data are presented as mean ± standard deviation. Statistical differences were assessed using analysis of variance (ANOVA: Tukey’s honestly significant difference test) to perform a comparison on all three groups. For analyzing the relationship between medication selected as first rank and background factor (continuous scale), ANOVA was performed to examine the difference among the three groups; Tukey's honestly significant difference test was used to estimate the difference between each group. For analyzing the relationship between medication selected as first rank and background factor (nominal scale), a χ2 test was performed using a contingency table. Statistical analyses were performed using JMP® version 14.2.0 statistical software (SAS Institute Japan, Tokyo, Japan). A difference with a *P* value (*P*) < 0.05 was considered statistically significant.

## Results

Seventy-five subjects were initially enrolled. The clinical characteristics of these patients are shown in Table 1. The population was 92% male, and included four current smokers. The mean percentage post-bronchodilator forced expiratory volume in one second (% post-BD FEV1) was 58.8%. Details of prior treatments are shown in Additional file 1: Table S2. As a prior therapeutic drug, LAMAs were used in 61 cases (81.3%); Tio was the most prescribed, and was used in 31 cases (50.8%). As a prior therapeutic drug, LABAs were used in 63 cases (84.0%); Ind was the most prescribed, and was used in 38 cases (60.3%). In addition, ICS were used in 25.3% cases; Ciclesonide was the most prescribed (52.6% of cases). Prior to the study, LAMA/LABA FDCs were used in 27 cases (Gly/Ind, 19 cases; Ume/Vil, four cases; Tio/Olo, four cases).

**Table 1:** Baseline clinical characteristics of patients

N	75	IC (L)	1.91 ± 0.51
Male:female	69:6	FVC (L)	2.81 ± 0.72
Age (years)	77.4 ± 6.9 (53–91)	FEV1 (L)	1.42 ± 0.54
Height (cm)	164.0 ± 7.2	FEV1/FVC (%)	50.5 ± 13.5
Body weight (kg)	59.3 ± 9.8	MMF (L/s)	0.56 ± 0.35
BMI (kg/m^2^)	22.0 ± 3.0	PF (L/s)	4.37 ± 1.72
Smoking (pack year)	Former: 71	V50 (L/s)	0.71 ± 0.47
	Current: 4	V25 (L/s)	0.25 ± 0.14
	52.6 ± 26.8	V50/V25	2.87 ± 1.06
*PIF* (L/min)			
Adaptor-free	195.1 ± 62.3	Post-BD FEV1 (L)	1.46 ± 0.54
H/H	42.4 ± 7.4	%post-BD FEV1 (%)	58.8 ± 21.7
Asthma	8	Severity of airflow obstruction (grade)	I: 14
			II: 36
			III: 16
			IV: 9

BMI, body mass index; PIF, peak inspiratory flow, H/H, adaptor for handihaler; IC, inspiratory capacity; FVC, forced vital capacity; FEV1, forced expiratory volume in one second; MMF, maximal mid-expiratory flow; PF, peak expiratory flow; V50, forced expiratory flow at 50% of FVC; V25, forced expiratory flow at 25% of FVC; BD, bronchodilator

The trial profile is shown in Fig. 1. Enrolled subjects were randomly divided into three groups: Gly/Ind first group; Ume/Vil first group, or Tio/Olo first group. Four cases were not included in the final analysis of our study: two cases dropped out due to the influences of other diseases; one case dropped out with exacerbation; one case did not complete the return visit to the hospital. Therefore, in total, 71 cases were analyzed. All subjects analyzed were investigated at 4 weeks without any postponement.

According to the questionnaire surveys completed at the end of each medication, no significant difference in CAT score or mMRC score was reported between each group (Table 2). In total, 11 adverse events were recorded in our original questionnaire. The Ume/Vil score for “Aftertaste” was significantly higher than the corresponding Gly/Ind or Tio/Olo scores. However, the reported scores for all of the adverse events were low; less than one (Table 2). When the mean scores of the “Difficulty of urination” item (that supposed prostatic hypertrophy) were evaluated only in females, the results were 0 ± 0 for Gly/Ind, 0.80 ± 1.79 for Ume/Vil, and 0.60 ± 1.34 for Tio/Olo; no significant difference was observed between any of the groups. Moreover, the scores were comparable to the scores observed in other items. Therefore, these low scores could not be considered to be clinically significant adverse events. Finally, there were no cases of discontinuation of medications due to adverse events.

**Table 2:** Scores on CAT, mMRC, and original questionnaire survey, and visual analog scale after using each inhaler

	Gly/Ind	Ume/Vil	Tio/Olo	*P* value		
				Gly/Ind versus Ume/Vil	Gly/Ind versus Tio/Olo	Ume/Vil versus Tio/Olo
CAT	14.2 ± 8.5	14.6 ± 9.0	14.1 ± 8.6	0.9454	0.9975	0.9208
mMRC	1.86 ± 0.95	1.80 ± 1.01	1.89 ± 0.95	0.9359	0.9836	0.8617
*Original questionnaire*						
① Hoarseness	0.39 ± 0.84	0.31 ± 0.67	0.28 ± 0.54	0.7473	0.5964	0.9681
② Discomfort or irritation of the throat	0.39 ± 0.62	0.51 ± 0.91	0.27 ± 0.51	0.6027	0.5273	0.1051
③ Cough immediately after inhalation	0.54 ± 0.69	0.56 ± 0.86	0.46 ± 0.75	0.9742	0.8495	0.7267
④ Aftertaste	0.31 ± 0.60	0.65 ± 0.83	0.23 ± 0.45	*0.0059*	0.7164	*0.0004*
⑤ Headache	0.10 ± 0.45	0.08 ± 0.41	0.10 ± 0.34	0.9764	1	0.9764
⑥ Palpitation	0.14 ± 0.35	0.20 ± 0.52	0.08 ± 0.33	0.692	0.692	0.2325
⑦ Tremor	0.06 ± 0.23	0.10 ± 0.34	0.08 ± 0.28	0.6597	0.8309	0.9547
⑧ Eye pain, bleariness	0.20 ± 0.52	0.34 ± 0.70	0.32 ± 0.63	0.3671	0.4436	0.9899
⑨ Thirst	0.59 ± 0.87	0.53 ± 0.84	0.46 ± 0.71	0.8633	0.622	0.9102
⑩ Constipation	0.32 ± 0.63	0.46 ± 0.77	0.30 ± 0.62	0.4303	0.9666	0.2981
⑪ Difficulty of urination	0.30 ± 0.54	0.31 ± 0.71	0.28 ± 0.68	0.9908	0.9908	0.9638
⑫ About shape, size or design	1.01 ± 0.62	1.42 ± 0.77	1.21 ± 0.75	*0.0024*	0.2323	0.1878
⑬ About inhaler operation and procedure	1.06 ± 0.63	1.23 ± 0.74	1.14 ± 0.68	0.3082	0.7435	0.7435
⑭ About actual feeling of being able to inhale	1.01 ± 0.73	1.45 ± 1.01	1.64 ± 0.90	*0.0103*	*< 0.0001*	0.3821
⑮ About actual effect	1.58 ± 0.80	1.80 ± 0.77	1.70 ± 0.74	0.1936	0.5919	0.7277
⑯ Overall evaluation	1.35 ± 0.79	1.70 ± 0.93	1.54 ± 0.73	*0.0311*	0.3836	0.4416
Visual Analog Scale (cm)	6.65 ± 2.21	6.09 ± 2.32	6.06 ± 2.10	0.2924	0.2514	0.9954

CAT, COPD Assessment Test; mMRC, modified Medical Research Council dyspnea questionnaire

The rankings for “Shape, size, or design” evaluation for Gly/Ind were significantly superior compared to Ume/Vil. For the item “Actual feeling of being able to inhale”, Gly/Ind performed significantly better than Ume/Vil and Tio/Olo. In addition, Gly/Ind performed significantly better than Ume/Vil in the item “Overall evaluation” (Table 2).

The results of the respiratory function test are reported in Table 3. For spirometry and FOT, there were no significant differences in all items. In the expiratory phase, inspiratory phase, and expiratory phase minus inspiratory phase, there were also no significant differences for all FOT items (Additional file 1: Table S3).

**Table 3:** Spirometry and FOT by MostGraph-01® after using each inhaler

	Gly/Ind	Ume/Vil	Tio/Olo	*P* value		
				Gly/Ind versus Ume/Vil	Gly/Ind versus Tio/Olo	Ume/Vil versus Tio/Olo
*Spirometry*						
IC (L)	2.02 ± 0.54	2.03 ± 0.57	2.04 ± 0.56	0.9939	0.9544	0.9812
FVC (L)	2.87 ± 0.74	2.88 ± 0.72	2.95 ± 0.75	0.9993	0.8137	0.8332
FEV1 (L)	1.52 ± 0.57	1.51 ± 0.56	1.52 ± 0.56	0.9969	0.9999	0.9977
MMF (L/s)	0.64 ± 0.38	0.62 ± 0.36	0.61 ± 0.38	0.9620	0.8849	0.9769
PF (L/s)	4.77 ± 1.92	4.73 ± 1.78	4.76 ± 1.83	0.9891	0.9991	0.9944
V50 (L/s)	0.83 ± 0.52	0.82 ± 0.53	0.80 ± 0.56	0.9908	0.9457	0.9805
V25 (L/s)	0.29 ± 0.15	0.27 ± 0.13	0.26 ± 0.15	0.6932	0.6318	0.9947
V50/V25	2.90 ± 1.11	2.97 ± 1.18	2.97 ± 1.22	0.9279	0.9187	0.9997
*FOT (average)*						
R5 (cmH2O/L/s)	3.35 ± 1.11	3.38 ± 1.11	3.30 ± 1.19	0.9856	0.9568	0.8966
R20 (cmH2O/L/s)	2.58 ± 0.85	2.65 ± 0.85	2.52 ± 0.81	0.8771	0.9049	0.6321
R5–R20 (cmH2O/L/s)	0.78 ± 0.42	0.74 ± 0.40	0.78 ± 0.50	0.8418	0.9997	0.8288
X5 (cmH2O/L/s)	− 1.25 ± 1.12	− 1.25 ± 1.10	− 1.32 ± 1.31	0.9997	0.9413	0.9335
Fres (Hz)	12.75 ± 4.88	12.67 ± 4.88	12.85 ± 5.28	0.9952	0.9923	0.9754
ALX (cmH2O/L)	8.50 ± 10.18	8.44 ± 9.78	9.39 ± 12.39	0.9993	0.8781	0.8611

FOT, forced oscillation technique; IC, inspiratory capacity; FVC, forced vital capacity; FEV1, forced expiratory volume in one second; MMF, maximal mid-expiratory flow; PF, peak expiratory flow; V_50_, forced expiratory flow at 50% of FVC; V_25_, forced expiratory flow at 25% of FVC; R5, resistance of respiratory system at 5 Hz; R20, resistance of respiratory system at 20 Hz; X5, reactance of respiratory system at 5 Hz; Fres, resonant frequency; ALX, low-frequency reactance area

**Fig. 2:**
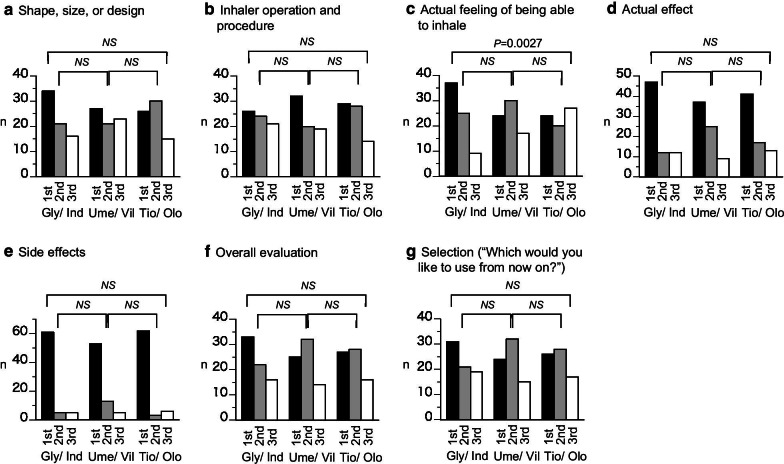
End of study Questionnaire. Selection and ranking of medications by patients. **a** Shape, size, or design; **b** Inhaler operation and procedure; **c** Actual feeling of being able to inhale; **d** Actual effect; **e** Side effects; **f** Overall evaluation; **g** Selection (“Which would you like to use from now on?”)

In the end of study comparative questionnaire, the ranking of “Actual feeling of being able to inhale” was significantly better for Gly/Ind compared with Tio/Olo;, Gly/Ind also tended to be better than Ume/Vil, although there was no significant difference (*P* = 0.0618). No significant differences were reported in the ranking of all other questionnaire items (Fig. 2). Regarding the reasons provided for selection preference (Additional file 1: Table S4), 57 subjects responded to free comments (14 did not respond). The reasons provided for selecting Gly/Ind included “a good feeling of being able to inhale due to transparent capsule or sound during inhalation” in 16 subjects and “good effects” in seven subjects. Ten subjects preferred Ume/Vil because of its easy operability. Reasons provided for preferring Tio/Olo included “Good effects” (four subjects) and the “Actual feeling of inhalation” (four subjects). One subject evaluated all three agents as equivalent.

Finally, the relationship between the medication selected as the first rank in the final comparative questionnaire and the background factors of the patients (sex, age, height, weight, BMI, PIF, respiratory function, presence or absence of asthma complication, types of inhalation device at baseline, presence or absence of ICS use, and order of LAMA/LABA FDCs) were investigated (Additional file 1: Table S5). However, no significant difference in the relationship between the first-ranking LAMA/LABA FDC and all background factors was observed.

## Discussion

Although the comparative efficiency and safety of different LAMA/LABA FDCs were previously reported in an indirect study (Tio/Olo ≫ Ume/Vil > Gly/Ind) [13], published findings concerning a direct comparison of Tio/Olo, Ume/Vil, and Gly/Ind are limited. These three LAMA/LABA FDCs were previously found to have similar incremental cost-effectiveness ratios [16], efficacy, and safety [17]. In a comparison of two medications, Ume/Vil was reported to be superior to Tio/Olo in rescue medication use and medication adherence [18], and was also reported to show better cost-effectiveness (compared to Tio/Olo) [19]. In a direct comparison test, Ume/Vil was superior to Tio/Olo regarding the change of trough FEV1 [14, 15]. However, taken together, the published reports appear to be contradictory. Moreover, the previous reports are limited direct comparison studies between two medications, retrospective studies, or indirect studies of the three medications. Therefore, to conclusively determine LAMA/LABA FDC efficacy, safety, and preference, we decided to conduct a direct comparison study of all three medications. This is the first prospective direct comparative study (open-labeled) among three once-daily LAMA/LABA FDCs.

The peak inspiratory flow (PIF) value is an important manifestation of the effects of inhaled drugs [5]. In this study, a Handihailer® adapter, a device with high intrinsic airflow resistance [20, 21], was used to measure PIF (the PIF meter adapter for Ellipta® had not been released at the start of this study). From the PIF levels, we determined that Ellipta® can be inhaled by most patients. No significant difference in respiratory functions was reported in this study, and no significant difference was also observed in CAT, mMRC, or in the actual effect (as reported in questionnaire surveys after each inhaler use and at the end of the study).

Although Gly/Ind was rated the best on “Actual feeling of being able to inhale” and superior to Ume/Vil on shape and overall evaluation in the survey after using each inhaler, Gly/Ind seemed to be preferred only on “Actual feeling of being able to inhale” in the final comparison survey (at the end of study). Although the reason for this preference was unknown, the transparent inhalation capsule or the sound produced during inhalation, may explain the inhalation feeling reported in the free comments.

In the final comparison survey, Ume/Ind was ranked first more times than the other medications for inhaler operation and procedure. However, no significant difference was reported among the three medications. In the free comments, ten subjects reported ease of operability as the reason for their stated preference. It should be noted that Ellipta has a higher correct use rate and a lower error rate than MDI [22].

Respimat® demonstrated the lowest amount of particles deposited in a mouth-throat model, and the highest amount of particles reaching all regions of the simulation lung model (compared to Breezhaler® and Ellipta®) [23]. Unsurprisingly, feelings of discomfort or irritation in the throat reported in the survey after 4 weeks treatment with each medication were lowest for Tio/Olo, although there were no significant differences.

Regarding the safety of each medication [4, 24–26], the scores of side effects reported in the questionnaire survey were less than one after each medication; there were no significant differences except for “Aftertaste”. There were also no withdrawals due to adverse events. Therefore, it was concluded that the tolerability of each medication was good. According to recent reports, LAMA/LABA combination therapy may improve left ventricular end-diastolic volume, possibly by improving lung hyperinflation [27]. Moreover, LAMA/LABA combination therapy may improve cardiac function in COPD patients with heart failure [28].

As important limitations of this study, there is the fact that issues regarding inhaler device handling were not accounted for. Although inhaler device handling is undoubtedly important [29], we could not confirm whether or not inhalation was always successful. Nonetheless, demonstration is the most effective means for device operation instruction [30], and handling instructions were provided through both demonstrations and written instructions. Secondly, washing out periods could not be provided due to the real world clinical setting. Thirdly, the number of patients who used Gly/Ind before this study was higher than that of Ume/Vil or Tio/Olo users (19 subjects vs. 4 subjects in other FDCs). This difference might have influenced the preferences such as “Actual feeling of being able to inhale”. Finally, compared to global reports, the proportion of women with COPD in Japanese COPD studies is small (8.0–14.5%) [31–33]; the proportion of women in this study was also small (8.0%). These differences in sex ratios between various countries might have influenced results such as preference.

## Conclusions

Except for the “Actual feeling of being able to inhale”, there was no definitive clinical difference among the three LAMA/LABA FDCs. Moreover, the safety profiles of all three LAMA/LABA FDCs were very good. Although the characteristics of patients who expressed a preference for one of the three medications were investigated, characteristic factors that predicted which medication was preferred could not be found.

## Supplementary information

**Additional file 1: Figure S1**. End of the study comparative questionnaire concerning all three medications. **Table S1**. Original questionnaire for completion after using each inhaler. **Table S2**. Treatments used prior to this study. **Table S3**. Forced oscillation technique (FOT) at expiration phase, inspiration phase, and ΔExpiration minus inspiration phase using MostGraph-01® after each inhaler use. **Table S4**. Reasons for selection (in free comments). **Table S5**. Relationship between first-ranking LAMA/ LABA FDC and background factors of patients.

## Data Availability

The datasets used and/or analysed during the current study are available from the corresponding author on reasonable request.

## Abbreviations

ANOVA: Analysis of variance
CAT: COPD Assessment Test
COPD: Chronic obstructive pulmonary disease
FDC: Fixed-dose combination
FEV1: Forced expiratory volume in one second
Gly: Glycopyrronium
ICS: Inhaled corticosteroid
Ind: Indacaterol
LABA: Long-acting β2 agonist
LAMA: Long-acting muscarinic antagonist
mMRC: Modified Medical Research Council dyspnea questionnaire
Olo: Olodaterol
PIF: Peak inspiratory flow
Tio: Tiotropium
Ume: Umeclidinium
Vil: Vilanterol
