# Supplementary material for: A randomized controlled trial of long-acting muscarinic antagonist and long-acting β2 agonist fixed-dose combinations in patients with chronic obstructive pulmonary disease
Source: BMC Pulm Med. 2021 Jan 13;21:26. doi: 10.1186/s12890-021-01403-y (PMC7805049; doi:10.1186/s12890-021-01403-y)
Supplement: Supplementary file 1 — Additional file 1: Figure S1. End of the study comparative questionnaire concerning all three medications. Table S1. Original questionnaire for completion after using each inhaler. Table S2. Treatments used prior to this study. Table S3. Forced oscillation technique (FOT) at expiration phase, inspiration phase, and ΔExpiration minus inspiration phase using MostGraph-01® after each inhaler use. Table S4. Reasons for selection (in free comments). Table S5. Relationship between first-ranking LAMA/ LABA FDC and background factors of patients. [file 12890_2021_1403_MOESM1_ESM.pptx]

## Slide 1
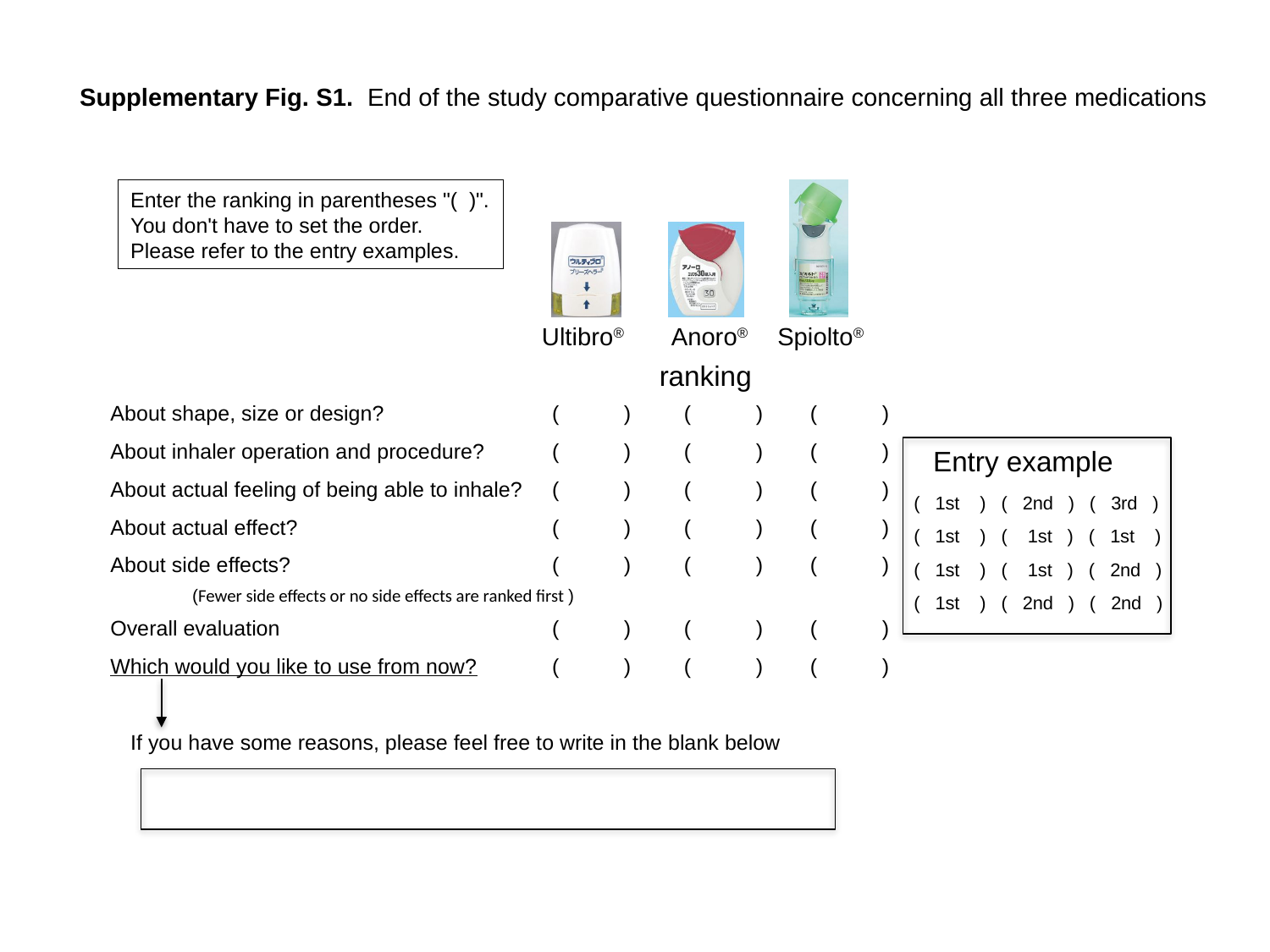

Supplementary Fig. S1. End of the study comparative questionnaire concerning all three medications
Enter the ranking in parentheses "( )".
You don't have to set the order.
Please refer to the entry examples.
Ultibro®
Anoro®
Spiolto®
ranking
About shape, size or design?
About inhaler operation and procedure?
About actual feeling of being able to inhale?
About actual effect?
About side effects?
Overall evaluation
Which would you like to use from now?
( ) ( ) ( )
( ) ( ) ( )
( ) ( ) ( )
( ) ( ) ( )
( ) ( ) ( )
( ) ( ) ( )
( ) ( ) ( )
Entry example
( 1st ) ( 2nd ) ( 3rd )
( 1st ) ( 1st ) ( 1st )
( 1st ) ( 1st ) ( 2nd )
( 1st ) ( 2nd ) ( 2nd )
(Fewer side effects or no side effects are ranked first )
If you have some reasons, please feel free to write in the blank below

## Slide 2
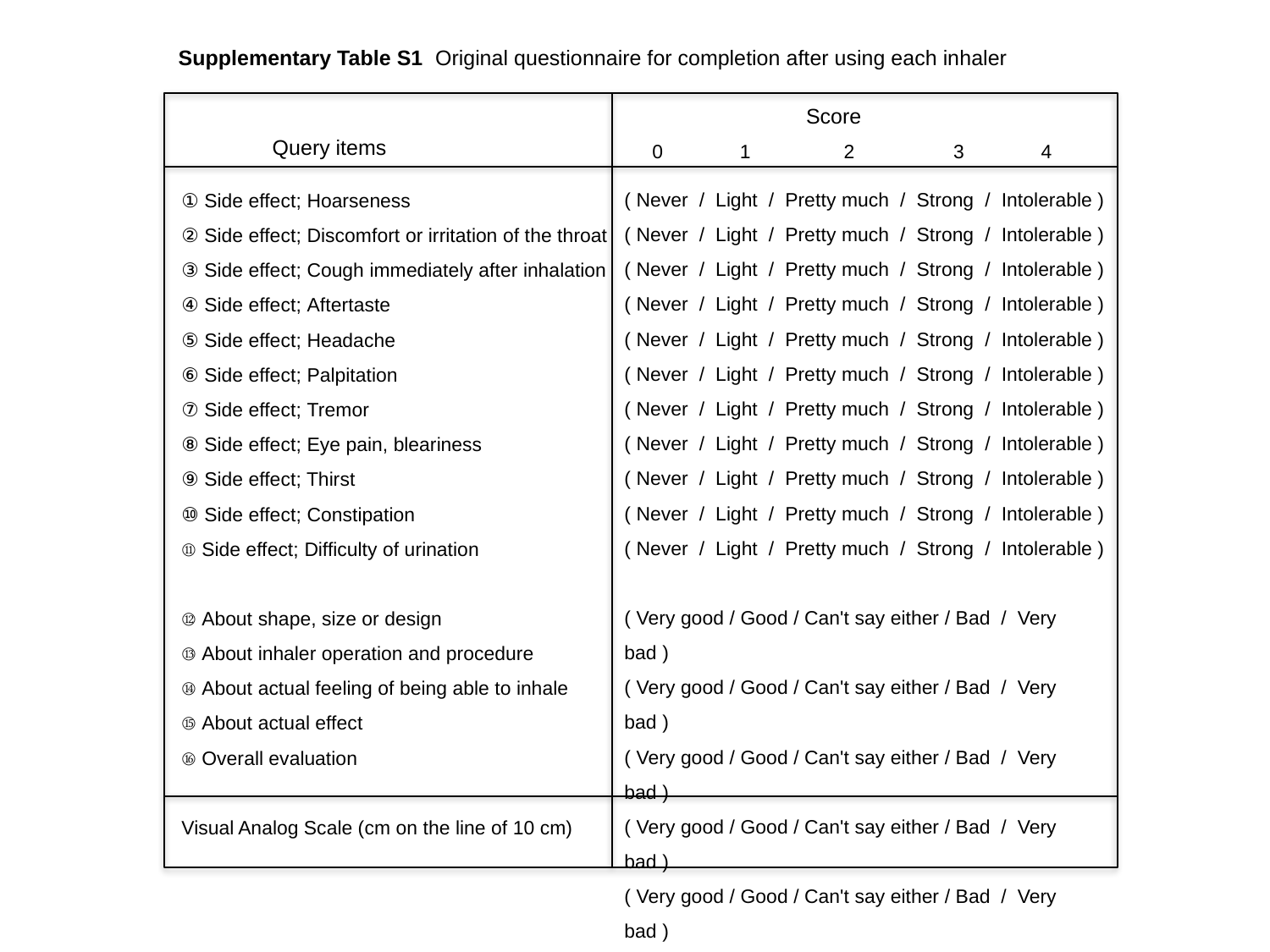

Supplementary Table S1 Original questionnaire for completion after using each inhaler
Score
Query items
0 1 2 3 4
( Never / Light / Pretty much / Strong / Intolerable )
( Never / Light / Pretty much / Strong / Intolerable )
( Never / Light / Pretty much / Strong / Intolerable )
( Never / Light / Pretty much / Strong / Intolerable )
( Never / Light / Pretty much / Strong / Intolerable )
( Never / Light / Pretty much / Strong / Intolerable )
( Never / Light / Pretty much / Strong / Intolerable )
( Never / Light / Pretty much / Strong / Intolerable )
( Never / Light / Pretty much / Strong / Intolerable )
( Never / Light / Pretty much / Strong / Intolerable )
( Never / Light / Pretty much / Strong / Intolerable )
( Very good / Good / Can't say either / Bad / Very bad )
( Very good / Good / Can't say either / Bad / Very bad )
( Very good / Good / Can't say either / Bad / Very bad )
( Very good / Good / Can't say either / Bad / Very bad )
( Very good / Good / Can't say either / Bad / Very bad )
① Side effect; Hoarseness
② Side effect; Discomfort or irritation of the throat
③ Side effect; Cough immediately after inhalation
④ Side effect; Aftertaste
⑤ Side effect; Headache
⑥ Side effect; Palpitation
⑦ Side effect; Tremor
⑧ Side effect; Eye pain, bleariness
⑨ Side effect; Thirst
⑩ Side effect; Constipation
⑪ Side effect; Difficulty of urination
⑫ About shape, size or design
⑬ About inhaler operation and procedure
⑭ About actual feeling of being able to inhale
⑮ About actual effect
⑯ Overall evaluation
Visual Analog Scale (cm on the line of 10 cm)

## Slide 3
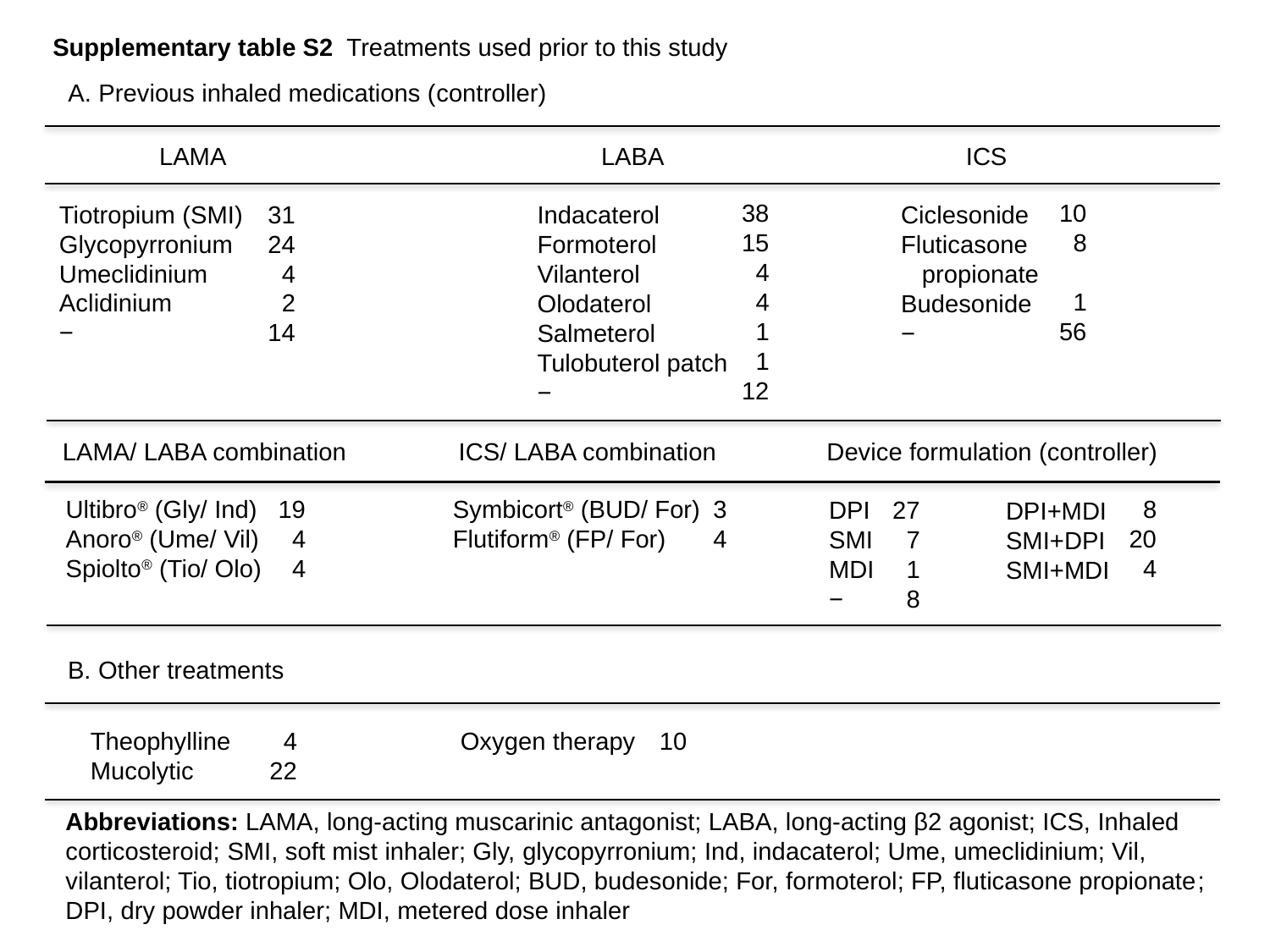

Supplementary table S2 Treatments used prior to this study
A. Previous inhaled medications (controller)
LAMA
LABA
ICS
38
15
 4
 4
 1
 1
12
10
 8
 1
56
Tiotropium (SMI)
Glycopyrronium
Umeclidinium
Aclidinium
−
31
24
 4
 2
14
Indacaterol
Formoterol
Vilanterol
Olodaterol
Salmeterol
Tulobuterol patch
−
Ciclesonide
Fluticasone
 propionate
Budesonide
−
LAMA/ LABA combination
ICS/ LABA combination
Device formulation (controller)
Ultibro® (Gly/ Ind)
Anoro® (Ume/ Vil)
Spiolto® (Tio/ Olo)
19
 4
 4
Symbicort® (BUD/ For)
Flutiform® (FP/ For)
 3
 4
 8
20
 4
27
 7
 1
 8
DPI
SMI
MDI
−
DPI+MDI
SMI+DPI
SMI+MDI
B. Other treatments
Theophylline
Mucolytic
 4
22
Oxygen therapy
10
Abbreviations: LAMA, long-acting muscarinic antagonist; LABA, long-acting β2 agonist; ICS, Inhaled corticosteroid; SMI, soft mist inhaler; Gly, glycopyrronium; Ind, indacaterol; Ume, umeclidinium; Vil, vilanterol; Tio, tiotropium; Olo, Olodaterol; BUD, budesonide; For, formoterol; FP, fluticasone propionate; DPI, dry powder inhaler; MDI, metered dose inhaler

## Slide 4
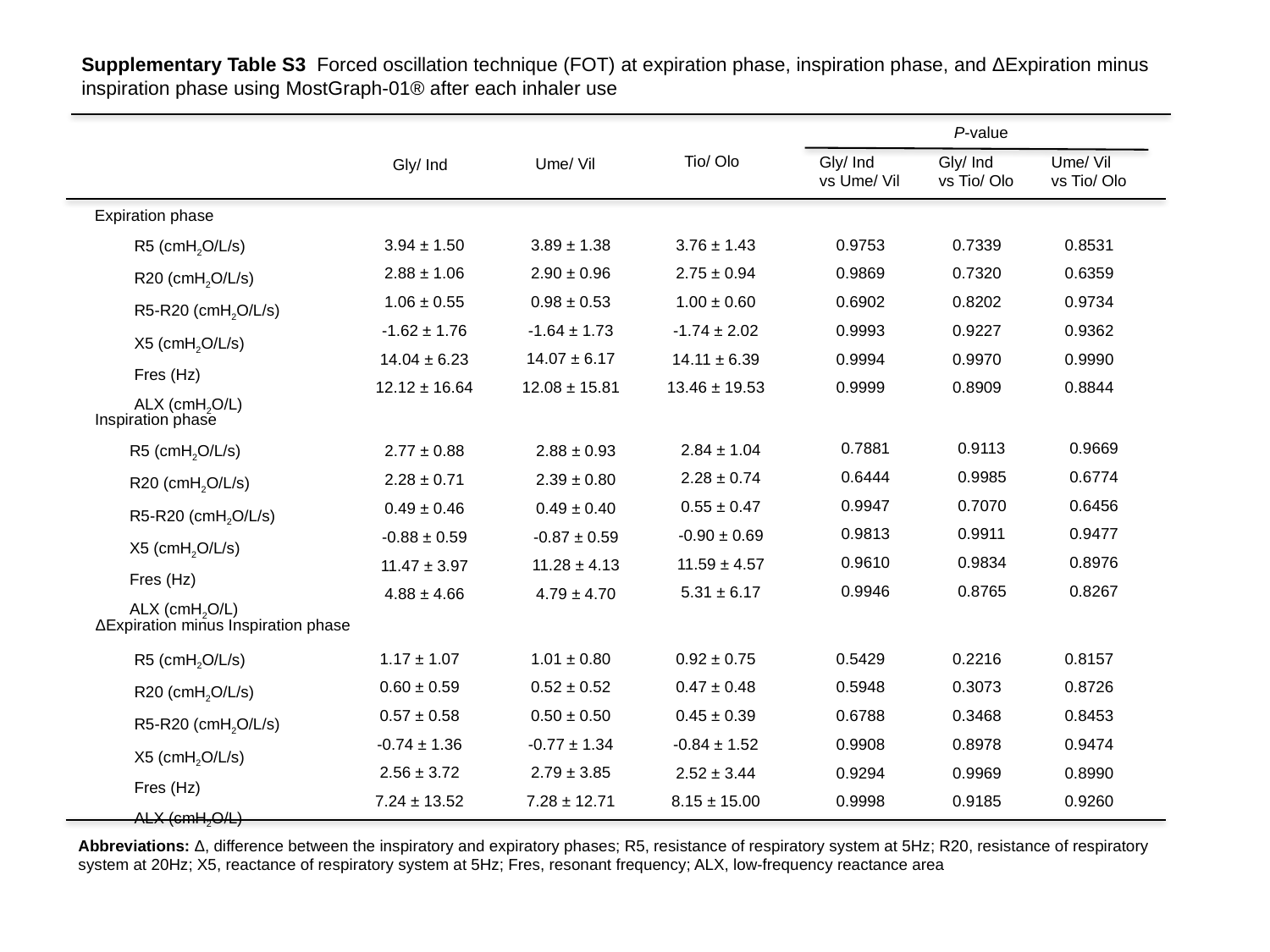

Supplementary Table S3 Forced oscillation technique (FOT) at expiration phase, inspiration phase, and ΔExpiration minus inspiration phase using MostGraph-01® after each inhaler use
P-value
Tio/ Olo
Gly/ Ind
vs Ume/ Vil
Gly/ Ind
vs Tio/ Olo
Ume/ Vil
vs Tio/ Olo
Ume/ Vil
Gly/ Ind
Expiration phase
R5 (cmH2O/L/s)
R20 (cmH2O/L/s)
R5-R20 (cmH2O/L/s)
X5 (cmH2O/L/s)
Fres (Hz)
ALX (cmH2O/L)
3.89 ± 1.38
2.90 ± 0.96
0.98 ± 0.53
-1.64 ± 1.73
14.07 ± 6.17
12.08 ± 15.81
3.94 ± 1.50
2.88 ± 1.06
1.06 ± 0.55
-1.62 ± 1.76
14.04 ± 6.23
12.12 ± 16.64
3.76 ± 1.43
2.75 ± 0.94
1.00 ± 0.60
-1.74 ± 2.02
14.11 ± 6.39
13.46 ± 19.53
0.9753
0.9869
0.6902
0.9993
0.9994
0.9999
0.7339
0.7320
0.8202
0.9227
0.9970
0.8909
0.8531
0.6359
0.9734
0.9362
0.9990
0.8844
Inspiration phase
0.7881
0.6444
0.9947
0.9813
0.9610
0.9946
0.9113
0.9985
0.7070
0.9911
0.9834
0.8765
0.9669
0.6774
0.6456
0.9477
0.8976
0.8267
R5 (cmH2O/L/s)
R20 (cmH2O/L/s)
R5-R20 (cmH2O/L/s)
X5 (cmH2O/L/s)
Fres (Hz)
ALX (cmH2O/L)
2.84 ± 1.04
2.28 ± 0.74
0.55 ± 0.47
-0.90 ± 0.69
11.59 ± 4.57
5.31 ± 6.17
2.88 ± 0.93
2.39 ± 0.80
0.49 ± 0.40
-0.87 ± 0.59
11.28 ± 4.13
4.79 ± 4.70
2.77 ± 0.88
2.28 ± 0.71
0.49 ± 0.46
-0.88 ± 0.59
11.47 ± 3.97
4.88 ± 4.66
ΔExpiration minus Inspiration phase
R5 (cmH2O/L/s)
R20 (cmH2O/L/s)
R5-R20 (cmH2O/L/s)
X5 (cmH2O/L/s)
Fres (Hz)
ALX (cmH2O/L)
1.01 ± 0.80
0.52 ± 0.52
0.50 ± 0.50
-0.77 ± 1.34
2.79 ± 3.85
7.28 ± 12.71
1.17 ± 1.07
0.60 ± 0.59
0.57 ± 0.58
-0.74 ± 1.36
2.56 ± 3.72
7.24 ± 13.52
0.92 ± 0.75
0.47 ± 0.48
0.45 ± 0.39
-0.84 ± 1.52
2.52 ± 3.44
8.15 ± 15.00
0.5429
0.5948
0.6788
0.9908
0.9294
0.9998
0.2216
0.3073
0.3468
0.8978
0.9969
0.9185
0.8157
0.8726
0.8453
0.9474
0.8990
0.9260
Abbreviations: Δ, difference between the inspiratory and expiratory phases; R5, resistance of respiratory system at 5Hz; R20, resistance of respiratory system at 20Hz; X5, reactance of respiratory system at 5Hz; Fres, resonant frequency; ALX, low-frequency reactance area

## Slide 5
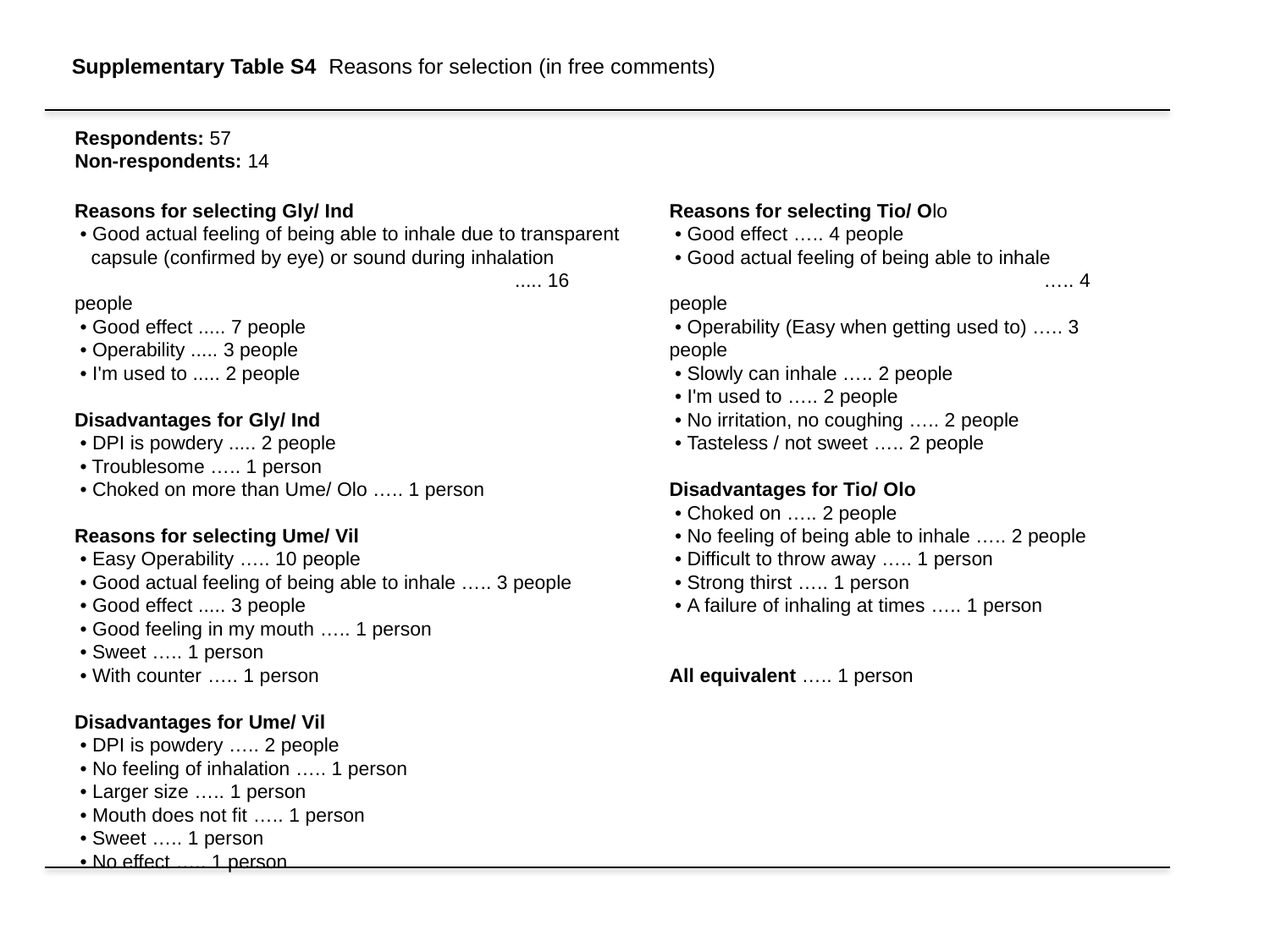

Supplementary Table S4 Reasons for selection (in free comments)
Respondents: 57
Non-respondents: 14
Reasons for selecting Gly/ Ind
 • Good actual feeling of being able to inhale due to transparent
 capsule (confirmed by eye) or sound during inhalation
 ..... 16 people
 • Good effect ..... 7 people
 • Operability ..... 3 people
 • I'm used to ..... 2 people
Disadvantages for Gly/ Ind
 • DPI is powdery ..... 2 people
 • Troublesome ….. 1 person
 • Choked on more than Ume/ Olo ….. 1 person
Reasons for selecting Ume/ Vil
 • Easy Operability ….. 10 people
 • Good actual feeling of being able to inhale ….. 3 people
 • Good effect ..... 3 people
 • Good feeling in my mouth ….. 1 person
 • Sweet ….. 1 person
 • With counter ….. 1 person
Disadvantages for Ume/ Vil
 • DPI is powdery ….. 2 people
 • No feeling of inhalation ….. 1 person
 • Larger size ….. 1 person
 • Mouth does not fit ….. 1 person
 • Sweet ….. 1 person
 • No effect ….. 1 person
Reasons for selecting Tio/ Olo
 • Good effect ….. 4 people
 • Good actual feeling of being able to inhale
 ….. 4 people
 • Operability (Easy when getting used to) ….. 3 people
 • Slowly can inhale ….. 2 people
 • I'm used to ….. 2 people
 • No irritation, no coughing ….. 2 people
 • Tasteless / not sweet ….. 2 people
Disadvantages for Tio/ Olo
 • Choked on ….. 2 people
 • No feeling of being able to inhale ….. 2 people
 • Difficult to throw away ….. 1 person
 • Strong thirst ….. 1 person
 • A failure of inhaling at times ….. 1 person
All equivalent ….. 1 person

## Slide 6
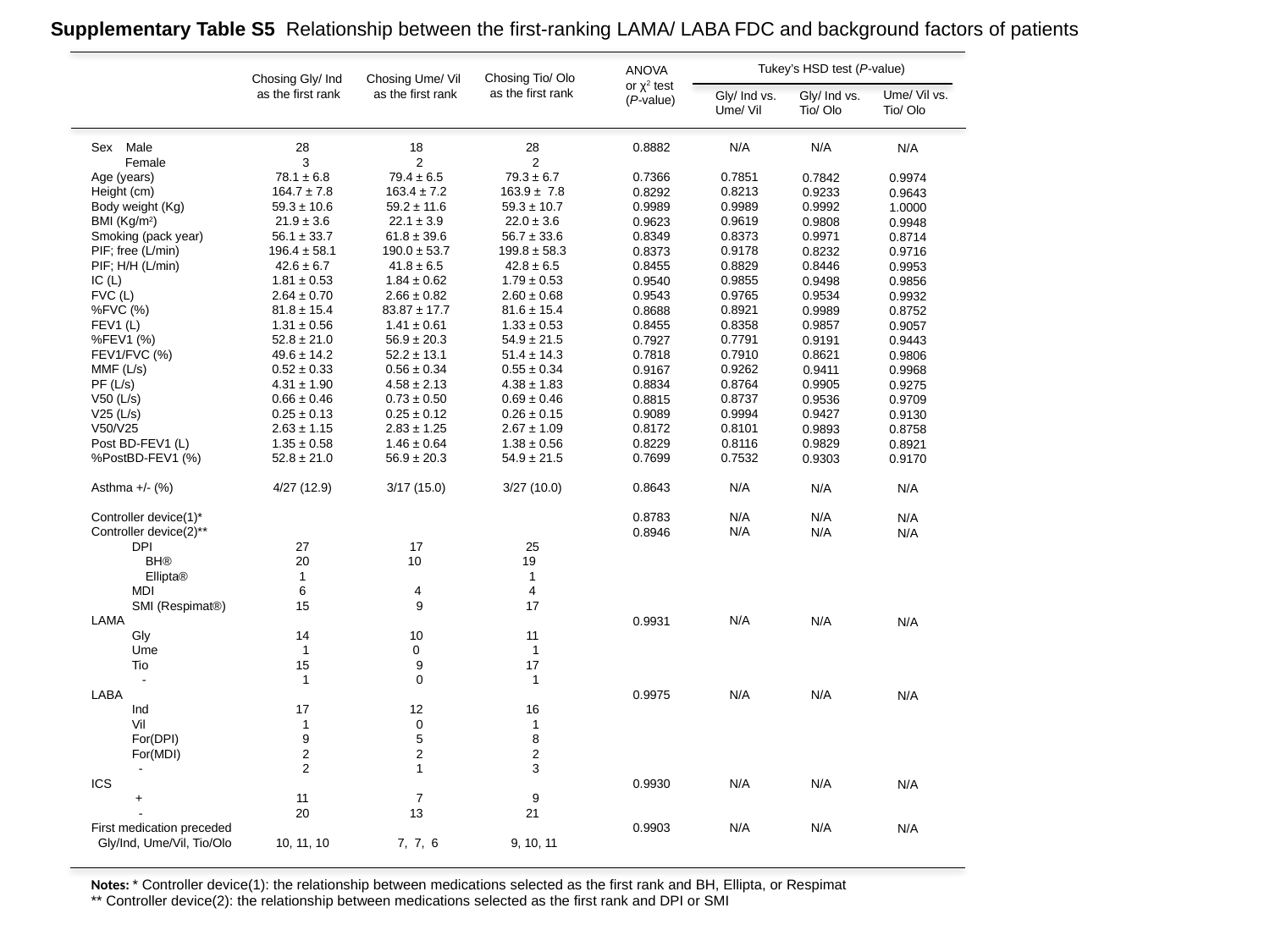

Supplementary Table S5 Relationship between the first-ranking LAMA/ LABA FDC and background factors of patients
Tukey’s HSD test (P-value)
ANOVA
or χ2 test
(P-value)
Chosing Tio/ Olo
as the first rank
Chosing Gly/ Ind
as the first rank
Chosing Ume/ Vil
as the first rank
Ume/ Vil vs.
Tio/ Olo
Gly/ Ind vs.
Tio/ Olo
Gly/ Ind vs.
Ume/ Vil
18
 2
79.4 ± 6.5
163.4 ± 7.2
59.2 ± 11.6
22.1 ± 3.9
61.8 ± 39.6
190.0 ± 53.7
41.8 ± 6.5
1.84 ± 0.62
2.66 ± 0.82
83.87 ± 17.7
1.41 ± 0.61
56.9 ± 20.3
52.2 ± 13.1
0.56 ± 0.34
4.58 ± 2.13
0.73 ± 0.50
0.25 ± 0.12
2.83 ± 1.25
1.46 ± 0.64
56.9 ± 20.3
3/17 (15.0)
17
10
 4
 9
10
 0
 9
 0
12
 0
 5
 2
 1
 7
13
 7, 7, 6
Sex Male
 Female
Age (years)
Height (cm)
Body weight (Kg)
BMI (Kg/m2)
Smoking (pack year)
PIF; free (L/min)
PIF; H/H (L/min)
IC (L)
FVC (L)
%FVC (%)
FEV1 (L)
%FEV1 (%)
FEV1/FVC (%)
MMF (L/s)
PF (L/s)
V50 (L/s)
V25 (L/s)
V50/V25
Post BD-FEV1 (L)
%PostBD-FEV1 (%)
Asthma +/- (%)
Controller device(1)*
Controller device(2)**
 DPI
 BH®
 Ellipta®
 MDI
 SMI (Respimat®)
LAMA
 Gly
 Ume
 Tio
 -
LABA
 Ind
 Vil
 For(DPI)
 For(MDI)
 -
ICS
 +
 -
First medication preceded
 Gly/Ind, Ume/Vil, Tio/Olo
28
 3
78.1 ± 6.8
164.7 ± 7.8
59.3 ± 10.6
21.9 ± 3.6
56.1 ± 33.7
196.4 ± 58.1
42.6 ± 6.7
1.81 ± 0.53
2.64 ± 0.70
81.8 ± 15.4
1.31 ± 0.56
52.8 ± 21.0
49.6 ± 14.2
0.52 ± 0.33
4.31 ± 1.90
0.66 ± 0.46
0.25 ± 0.13
2.63 ± 1.15
1.35 ± 0.58
52.8 ± 21.0
4/27 (12.9)
27
20
1
6
15
14
 1
15
 1
17
 1
 9
 2
 2
11
20
10, 11, 10
28
 2
79.3 ± 6.7
163.9 ± 7.8
59.3 ± 10.7
22.0 ± 3.6
56.7 ± 33.6
199.8 ± 58.3
42.8 ± 6.5
1.79 ± 0.53
2.60 ± 0.68
81.6 ± 15.4
1.33 ± 0.53
54.9 ± 21.5
51.4 ± 14.3
0.55 ± 0.34
4.38 ± 1.83
0.69 ± 0.46
0.26 ± 0.15
2.67 ± 1.09
1.38 ± 0.56
54.9 ± 21.5
3/27 (10.0)
25
19
1
4
17
11
 1
17
 1
16
 1
 8
 2
 3
 9
21
 9, 10, 11
N/A
0.7851
0.8213
0.9989
0.9619
0.8373
0.9178
0.8829
0.9855
0.9765
0.8921
0.8358
0.7791
0.7910
0.9262
0.8764
0.8737
0.9994
0.8101
0.8116
0.7532
N/A
N/A
N/A
N/A
N/A
N/A
N/A
0.8882
0.7366
0.8292
0.9989
0.9623
0.8349
0.8373
0.8455
0.9540
0.9543
0.8688
0.8455
0.7927
0.7818
0.9167
0.8834
0.8815
0.9089
0.8172
0.8229
0.7699
0.8643
0.8783
0.8946
0.9931
0.9975
0.9930
0.9903
N/A
0.7842
0.9233
0.9992
0.9808
0.9971
0.8232
0.8446
0.9498
0.9534
0.9989
0.9857
0.9191
0.8621
0.9411
0.9905
0.9536
0.9427
0.9893
0.9829
0.9303
N/A
N/A
N/A
N/A
N/A
N/A
N/A
N/A
0.9974
0.9643
1.0000
0.9948
0.8714
0.9716
0.9953
0.9856
0.9932
0.8752
0.9057
0.9443
0.9806
0.9968
0.9275
0.9709
0.9130
0.8758
0.8921
0.9170
N/A
N/A
N/A
N/A
N/A
N/A
N/A
Notes: * Controller device(1): the relationship between medications selected as the first rank and BH, Ellipta, or Respimat
** Controller device(2): the relationship between medications selected as the first rank and DPI or SMI
